# Selective, Stable and Humidity-Robust NO_2_ Sensing at Room Temperature with Porous WS_2_ Films

**DOI:** 10.1021/acssensors.5c02414

**Published:** 2025-10-27

**Authors:** Simone Hersberger, Michael Pereira Martins, Selina Fassbind, Andreas T. Güntner

**Affiliations:** Human-Centered Sensing Laboratory, Department of Mechanical and Process Engineering, 27219 ETH Zurich, CH-8092 Zurich, Switzerland

**Keywords:** nanotechnology, semiconductors, electronic materials, thin films, metal sulfides, gas sensors, environmental monitoring

## Abstract

Nitrogen dioxide (NO_2_) is a hazardous air pollutant with a lowered annual mean exposure limit from 40 to 10 μg/m^3^ (∼5 parts-per-billion by volume, ppb) by the World Health Organization in 2021. This motivates the exploration of low-power and cost-efficient sensors that can detect such low concentrations of NO_2_, exhibit high selectivity against interfering analytes and resilience to humidity fluctuations. Here, a selective, stable and humidity-robust sensor for NO_2_ sensing at room temperature is presented. Flame-aerosol deposition followed by dry sulfidation results in highly porous (98%) and nanostructured WS_2_ films. These films exhibit a 5-fold increase in response and over an order-of-magnitude reduction in response time, compared to conventional spin-coated films. Remarkable sensing performance down to 1 ppb of NO_2_ (with a signal-to-noise ratio of 12.9) is achieved with high selectivity (>164) toward environmental interferents including NH_3_, NO, acetone, H_2_S, benzene, CO, ethanol, methanol, N_2_O and toluene. We also reveal high robustness (response change ± 18%) against varying relative humidity (0–90%) and response stability over more than 6 months (±10%). This sensor outperforms previously reported NO_2_ sensors operating at room temperature, making it well-suited for integration into devices for environmental monitoring or wearables for personal exposure assessment.

## Graphical abstract

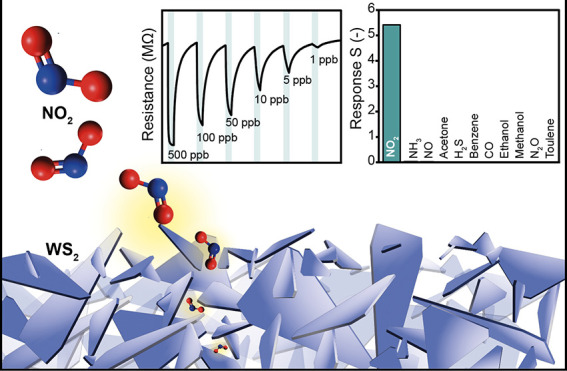

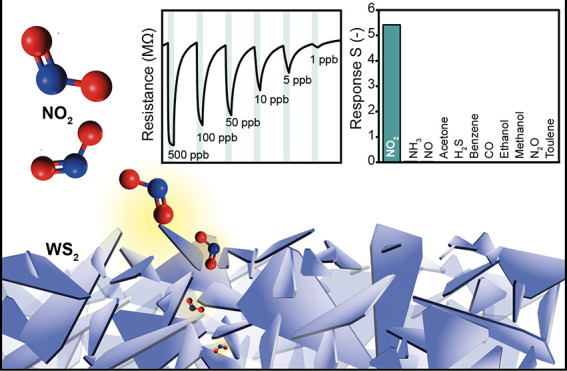

Nitrogen dioxide (NO_2_) is one of the most critical atmospheric pollutants, arising from fossil fuel combustion, biomass burning, power plants and industrial processes.^1^ Growing evidence links short^2^- and long-term^3^ exposure to elevated NO_2_ levels to respiratory and cardiovascular diseases. In response, the World Health Organization (WHO) has reduced the annual mean NO_2_ exposure limit from 40 to 10 μg/m^3^, the latter corresponding to ca. 5 parts-per-billion by volume (ppb) at standard conditions.^4^ To adhere to these public health guidelines, improved detectors are required, for instance, to enable large-scale monitoring networks. The standard method for NO_2_ quantification in air quality monitoring stations is chemiluminescence.^5^ This technique measures NO_2_ indirectly by first converting it to NO before quantifying the total NO concentration, rendering it prone to significant interference from other nitrogen-containing compounds.^6^ Furthermore, due to high maintenance and operating costs,^7^ these stations are sparsely deployed, resulting in limited spatial coverage, particularly in densely populated urban areas. Low-cost sensors offer a promising alternative for monitoring networks as well as for personal exposure tracking at workplaces with elevated NO_2_ levels. However, accurate detection at low ppb levels remains challenging due to various interferents and humidity fluctuations.

Chemoresistive sensors based on semiconductive metal oxide nanoparticles are known for their high sensitivity,^8^ miniaturization potential and low fabrication costs. To integrate these sensors into compact portable or wearable devices (e.g., protective clothes for personal exposure tracking^9^) and employ large-scale sensor networks, low power consumption is required. Several metal oxide sensors operated at moderate temperatures (50–150 °C) have been proposed for NO_2_ sensing, including In_2_O_3_ (50 °C) with a detection limit of 100 ppb,^10^ ZnO/rGO (130 °C) measuring down to 10 ppb^11^ and WO_3_ (125 °C) with detection down to 3 ppb.^8^ For continuous air quality monitoring, however, room temperature operation would further simplify device integration by eliminating the need for heating elements, enabling a more compact detector design with lower power consumption.

Various sensor material classes have been explored for room temperature NO_2_ detection, among them reduced graphene oxides modified with metal oxides^12–15^ and single-walled carbon nanotubes,^16^ due to their high specific surface area and strong charge transfer interaction with the analyte. However, the detection of concentrations close to the guideline values has not been demonstrated and selectivity to key confounding analytes in air remains unclear. Alternatively, two-dimensional transition metal dichalcogenides, including metal sulfides,^17^ offer narrow and tunable bandgaps,^18^ high surface reactivity and fast charge transfer^17^ at low temperature. A prominent metal sulfide for room temperature operation is WS_2_, which has been reported to detect down to 100 ppb^19^ of NO_2_ and shows selectivity over air quality-relevant interferents like NH_3_ and H_2_S.^20^ Yet, many of the reported metal sulfide-based sensors are limited by slow response and, in some cases, incomplete recovery.^19–22^ This may stem from the rather compact film structure, yielded by conventional wet-chemical synthesis methods (e.g., porosity of 48% for SnS_2_ fabricated by a hydrothermal method^23^), that mitigates effective mass transfer of analytes and reaction products. Overcoming this limitation requires alternative fabrication routes that yield porous and nanostructured films, which can facilitate rapid gas diffusion. However, such approaches have not yet been demonstrated for WS_2_, despite its promising intrinsic properties for room temperature NO_2_ sensing.

Here, a novel fabrication method is introduced to produce highly porous and nanostructured metal sulfide films for fast, selective and humidity-robust NO_2_ sensing at room temperature. Flame-aerosol synthesis^24^ was used to deposit WO_3_ nanoparticles onto alumina substrates with interdigitated electrodes. These porous films were dry-sulfidized while preserving the porous film architecture, as investigated by scanning electron microscopy (SEM) and X-ray diffraction (XRD). The effect of film morphology on NO_2_ sensing performance at room temperature was assessed by comparing these porous films to compact WS_2_ films of equal thickness obtained by spin-coating of equivalent WO_3_ nanoparticles followed by the same dry sulfidation process. The sensing performance of the porous films at room temperature was evaluated down to 1 ppb and tested for stability against various interfering analytes, robustness under varying relative humidity (RH) conditions and long-term stability over 6 months. To the best of our knowledge, this is the first demonstration of combined selective and humidity-robust NO_2_ sensing down to single ppb levels at room temperature using porous WS_2_ films.

## Methods

### Fabrication of WO_3_ Nanoparticles and Films

WO_3_ nanoparticles were prepared using flame-spray pyrolysis (FSP) with a reactor design introduced elsewhere.^25^ The liquid precursor was prepared by dissolving ammonium metatungstate hydrate (purity >99.99%, Sigma-Aldrich, Switzerland) in a 1:1 volumetric mixture of diethylene glycol monobutyl ether (>98%, Sigma-Aldrich, Switzerland) and ethanol (>99.8%, Sigma-Aldrich, Switzerland), yielding a metal concentration of 0.2 M.^26^ This solution was supplied to the FSP burner at a flow rate of 5 mL/min and dispersed by 5 L/min of O_2_ (5.0, Linde, Switzerland) into a fine spray at a pressure drop of 1.6 bar. A ring-shaped premixed flame consisting of O_2_ (3.25 L/min) and CH_4_ (1.25 L/min, 2.5, Linde, Switzerland) ignited and sustained the spray flame and an O_2_ sheath flow (5 L/min) was supplied from an annulus surrounding the flame. Porous films were produced by directly depositing^27^ WO_3_ nanoparticles for 2 min onto water-cooled Al_2_O_3_-based sensor substrates placed 20 cm above the burner. The sensor substrates (electrode type #103, Electronic Design Center, Case Western University, USA) contained interdigitated Pt electrodes (350 μm width and spacing) on the front and a Pt heater on the back. In addition, WO_3_ nanoparticles were collected on a water-cooled glass fiber filter (257 mm diameter, GF-6, Hahnemühle FineArt, Germany) positioned 57 cm above the burner using a vacuum pump. The nanoparticle powder was detached from the filter with a spatula and sieved through a 250 μm stainless steel mesh.

### Conversion of WO_3_ to WS_2_

The porous WO_3_ films were subsequently converted to WS_2_ via dry sulfidation with H_2_S. The WO_3_-loaded sensor substrates were placed inside a quartz tube with an inner diameter of 1.8 cm and bundles of glass wool (Sigma-Aldrich, Switzerland) were inserted up- and downstream of the sensor. The quartz tube was mounted into a tubular furnace (CTF 12/65/550, Carbolite, Germany) and heated to a target temperature of 500 °C, with a rate of 10 °C/min and a dwell time of 3 h. A flow of 80 mL/min N_2_ (4.5, Linde, Switzerland) was maintained during both heating and cooling using high-precision mass flow controllers (Bronkhorst, Netherlands). When the target temperature was reached, a flow of 20 mL/min of pure H_2_S (2.5, Linde, Switzerland) was supplied during the dwell time, while the N_2_ flow was maintained, to achieve an H_2_S concentration of 20%. Identical temperature and flow conditions were applied for the sulfidation of the WO_3_ powder in a quartz tube with an inner diameter of 1 cm. Glass wool was inserted up- and downstream of the powder with a small gap to avoid contamination.

For comparison, denser WS_2_ films were fabricated by spin-coating of the as-prepared FSP-made WO_3_ powder followed by the aforementioned dry sulfidation process. First, a suspension was prepared by mixing 8 mg of the WO_3_ powder with 180 μL of ethanol denatured with propan-2-ol (Alcosuisse AG, Switzerland). The particles were fully dispersed in the solvent by light ultrasonication (BANDELIN electronics GmbH & Co. KG, DT 106, Germany) for 30 s with rigorous shaking. This was followed by ultrasonication (Sonics & Materials Inc., VCX500, USA) for 3.5 min at 90% intensity under a constant flow of water and in cycles of 20 s followed by 5 s breaks, both to avoid heating of the suspension. The process was concluded with light ultrasonication and vigorous shaking. The as-obtained suspension was fully pipetted onto the sensor substrates. The sensor substrate was then spun (Laurell Technologies, WS-650MZ-23NPP/Lite, USA) for 15 min at 150 rpm with an acceleration of 50 rpm/s.

### Material Characterization

The crystallographic structure of the nanoparticle powders and films was characterized by XRD using a D2 Phaser diffractometer (Bruker, USA). The XRD patterns were acquired at 30 kV and 10 mA, at 2θ (Cu Kα radiation) between 10° and 70° with a scanning step size of 0.013° and a scanning rate of 1.1 s/step. The crystal phases were identified by comparison of the acquired patterns to the reference structural parameters of hexagonal WS_2_ (PDF 84-1398), cubic Pt (87-0647) and rhombohedral Al_2_O_3_ (48-0366) using the software Diffrac.eva V3 (Bruker, USA). Sample displacement was corrected by aligning the substrate-related Pt and Al_2_O_3_ diffraction peaks to their reference locations.

The film morphology was investigated using SEM with a cold field emission gun operated at an acceleration voltage of 3 kV and an emission current of 10 μA (Hitachi S-4800 FE-SEM, Hitachi High Tech, Japan). The obtained images were used to determine the deposited film thickness (*s*_sl_) from >100 measurements per produced film using the software ImageJ (USA), after cleaving the substrate for a cross-sectional image. The measured thickness was then used together with the attenuation of the X-ray signal of the Al_2_O_3_ peaks from the substrate, due to the deposited nanoparticle film,^27^ to determine the film porosity. Specifically, the peak intensity at 35.1, 43.3 and 57.5° of the bare (*I*_in_) and covered (*I*_em_) substrate were measured and the radiation pathway, *s*_s_*, determined using the exponential attenuation law^28^

$$\frac{I_{em}}{I_{in}}=\exp\left[-\frac{\mu }{\rho_{s}}\rho_{s}s_{s}^{*}\right]$$

with μ/ρ_s_ being the mass attenuation coefficient, which can be obtained from the XCOM Photon Cross Section Database,^29^ and ρ_s_ being the material density. The bulk film thickness (*s*_s_) was calculated by correcting the radiation pathway considering the incident angle and the double trajectory through the film. The porosity of the film was then determined by combining the deposited film thickness measured by SEM and the bulk film thickness calculated from XRD, with *V*_sl_ and *V*_s_ being the volume of the deposited and the bulk film, respectively

$$porosity=1-\frac{V_{s}}{V_{sl}}=1-\frac{s_{s}}{s_{sl}}$$

The porosity values reported correspond to the mean ± standard deviation calculated from the attenuation of the above-mentioned three Al_2_O_3_ XRD peaks. The elemental composition of the film was analyzed at 7 kV and 20 μA with X-ray energy-dispersive spectroscopy (EDXS) using an Ametek EDAX Octane Super Silicon Drift Detector mounted on the SEM.

High-resolution X-ray photoelectron spectroscopy (XPS) measurements were performed on as-prepared WO_3_ powders and dry-sulfidized WS_2_ powders. The binding energy scale was calibrated using the C 1s peak of adventitious carbon at 284.8 eV. The spectra were background-corrected using a Shirley function and fitted with mixed Gaussian–Lorentzian line shapes. For WO_3_, the O 1s region was fitted with lattice oxygen (529–530 eV), chemisorbed oxygen (∼531–532 eV) and physisorbed oxygen (∼533–536 eV). For WS_2_, only chemisorbed and physisorbed oxygen components were included. The full width at half-maximum was constrained to comparable values across components to avoid overfitting.

### Sensing Performance

The sensing performance of the WS_2_ films was evaluated by mounting the sensors onto a Macor holder and placing them into a Teflon chamber. Gas mixtures were prepared using a setup shown in Figure S1 and described in detail in a previous work.^32^ Briefly, dry-calibrated and certified gas standards were mixed with synthetic air (Linde, Switzerland, carbon-free grade: C_*n*_H_*m*_ and NO_*x*_ ≤ 100 ppb) using high-precision and calibrated mass flow controllers (Bronkhorst, Netherlands). The following gas standards (all from Linde, Switzerland, with synthetic air as balance gas) were used: NO_2_ (22.5 ppm), NH_3_ (10 ppm), NO (10.7 ppm), acetone (18.1 ppm), H_2_S (10.7 ppm), benzene (15 ppm), CO (506.2 ppm), ethanol (14.8 ppm), methanol (14.3 ppm), N_2_O (9.6 ppm) and toluene (15.9 ppm). Humidity was introduced into the analyte flow by bubbling dry synthetic air through deionized water at 22–23 °C. The sensing measurements were carried out at 50% RH, unless otherwise specified, and with a total gas flow of 1 L/min. The film resistance was monitored between the interdigitated Pt electrodes of the sensor substrate using a multimeter (Keithley, Integra Series 2700, USA). The lines of the setup were made of Teflon and heated to 55 °C to prevent water condensation and to minimize analyte adsorption.

Prior to each sensor measurement, the sensors were heated to 120 °C for 1 h under a synthetic air flow with 50% RH to clean the sensor surface. The measurements were started once the sensors reached a stable baseline after cooling down from the heat treatment. With WS_2_ exhibiting p-type semiconductor behavior, the response was defined as *S* = (*R*_air_/*R*_analyte_) – 1 for oxidizing analytes and as *S* = (*R*_analyte_/*R*_air_) – 1 for reducing analytes, with *R*_air_ and *R*_analyte_ corresponding to the steady state film resistances in synthetic air and during the exposure to the analyte, respectively. The response and recovery times were defined as the times required to reach or recover 90% of the resistance change, respectively. The selectivity was defined as the ratio between the response to NO_2_ and the response to an interfering analyte, both at identical concentration.

## Results and Discussion

### Fabrication and Characterization of Porous WS_2_ Films

For the design of porous WO_3_ films, flame-aerosol technology was utilized to form nanoparticle-based and fractal-like agglomerates through nucleation, coagulation and sintering followed by their thermophoretic deposition as fine networks.^24^ The highly porous film morphology of the obtained WS_2_ after dry sulfidation is confirmed by cross-sectional and top-view SEM images (Figure 1a). The film is composed of fine nanosheets, which are agglomerated to form hierarchically porous architectures featuring pores of varying size scales, as indicated in Figure 1a. The film exhibits a homogeneous morphology with a porosity of 98 ± 1% and an average thickness of 5.9 ± 0.5 μm (mean ± standard deviation). Note that film thicknesses at various deposition times are shown in Figure S2. This strategy for highly porous film fabrication through flame-aerosol deposition followed by dry conversion is rather flexible. The deposition can be applied to various other metal oxides (e.g., ZnO^33^) and even metastable nanocrystals (CoCu_2_O_3_ ^34^), with demonstrated subsequent conversion to metal bromides^35^ and nitrides.^36^

**Figure 1:**
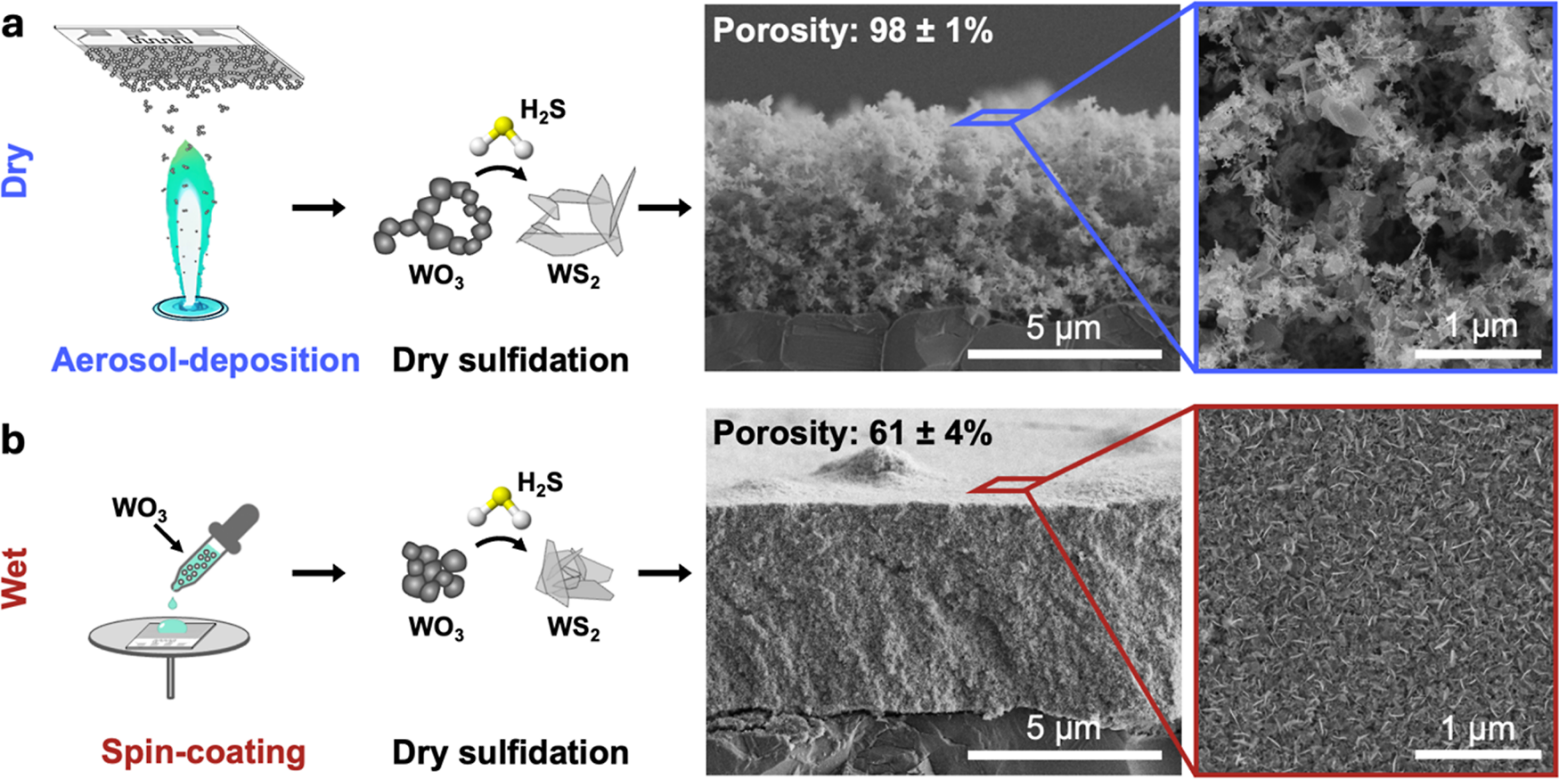
WS_2_ film fabrication. (a) WO_3_ nanoparticles are directly deposited onto water-cooled Al_2_O_3_ substrates by flame-aerosol deposition. Subsequently, dry sulfidation is performed at 20% H_2_S in N_2_ at 500 °C to produce porous WS_2_ films. Cross-sectional and top-view SEM images show the film thickness and film morphology with an average porosity of 98 ± 1%. (b) For comparison, compact films are produced by spin-coating of flame-made WO_3_ nanoparticles, which were collected on a glass fiber filter. Dry sulfidation of the spin-coated films is carried out under equal conditions resulting in a denser film morphology with an average porosity of 61 ± 4%.

Previously reported WS_2_ sensing films were fabricated by chemical vapor deposition,^37^ sputtering,^38^ thermal evaporation, hydrothermal synthesis^19^ or solution-based deposition methods. To have a direct comparison to a wet-deposition method, spin-coated films were prepared using the same flame-made WO_3_ nanoparticles (see Section Methods) followed by dry sulfidation with the film morphology being shown in Figure 1b. The resulting WS_2_ films exhibit a much denser morphology with a porosity of 61 ± 4% at a similar film thickness (5.9 ± 1.1 μm, note the same length scale in Figure 1a,b).

The dry-sulfidized WS_2_ is highly crystalline and consists exclusively of hexagonal nanocrystals (triangles, Figure 2a), as confirmed by the XRD pattern of the filter-collected powder. This is similar for the spin-coated films, where WS_2_-related peaks are clearly visible. Note that the Al_2_O_3_ (diamonds) and Pt (circles) peaks are attributed to the sensor substrate and its interdigitated electrodes, respectively. For the aerosol-deposited films, the WS_2_-related diffraction peaks are much weaker compared to those related to Al_2_O_3_, as expected for highly porous films due to their lower optical density. The most pronounced characteristic peaks of WS_2_ at 2θ ≈ 14.4° (Figure 2b), 32.8° and 33.6° (Figure 2c) are detectable and clearly distinguishable from those peaks associated with Al_2_O_3_ and Pt. No peak shift was observed for WS_2_-related peaks in the powder compared to spin-coated and aerosol-deposited films (Figure 2a–c).

**Figure 2:**
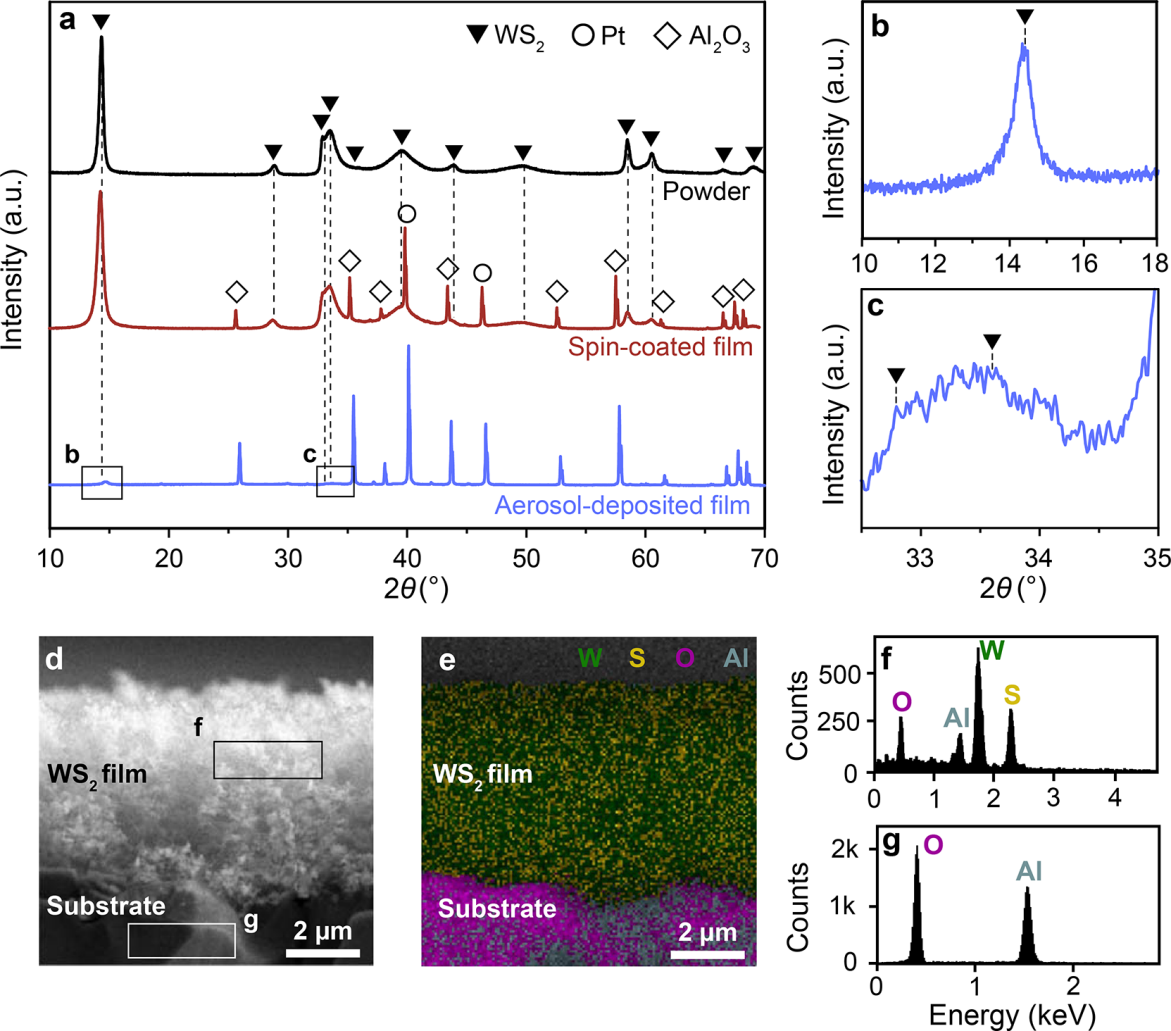
(a) XRD patterns of WS_2_ nanoparticle powder (black), a spin-coated WS_2_ film (red) and an aerosol-deposited WS_2_ film (blue) after dry sulfidation. Reference peak positions for hexagonal WS_2_ (triangles) are indicated. Note that the Al_2_O_3_ (diamonds) and Pt (circles) are associated with the sensor substrate and its interdigitated electrodes, respectively. Zoomed-in regions of the aerosol-deposited film at (b) 2θ = 10–18° and (c) 2θ = 32.5–35°. (d) Cross-sectional SEM image of the aerosol-deposited film and (e) corresponding elemental map with the distribution of W (green), S (yellow), O (pink) and Al (gray). EDXS spectra of (f) the WS_2_ film and (g) the sensor substrate, as indicated in (d), respectively. Note the different energy ranges in (f,g).

The elemental mapping of an aerosol-deposited and dry-sulfidized film’s cross-section (Figure 2d,e) reveals the primary presence of W (green) and S (yellow) in the film, while the substrate consists of O (pink) and Al (gray). This is confirmed by the corresponding EDXS spectrum of the film (Figure 2f), that shows strongest signals for W and S. Additional signals from Al and O likely originate from the underlying substrate, as the bare sensor substrate alone (Figure 2g) features a similar Al/O intensity ratio. Additionally, high-resolution O 1s XPS spectra were collected (Figure S3). The absence of the characteristic lattice oxygen signal from WO_3_ in the WS_2_ film suggests that the aerosol-deposited film has undergone complete sulfidation, in agreement with XRD (Figure 2a) and EDXS (Figure 2d–g).

### Effect of Film Morphology on NO_2_ Sensing Performance

High mass transfer in films is critical for various applications, including gas sensing.^39^ Therefore, we investigated the effect of the WS_2_ film morphology on the NO_2_ sensing performance at room temperature and at 50% RH. When the porous WS_2_ film is exposed to 1 ppm of NO_2_, the sensor shows p-type chemoresistive sensing behavior, i.e., the resistance decreases upon exposure to oxidative gases,^40^ with a response of 4.5 (Figure 3a: blue solid line and left ordinate). When evaluating six individually produced sensors at the same condition, the average response and standard deviation are 5.4 ± 1.2 (Figure 3b), indicating good reproducibility of the porous films. Most importantly, this response is five times higher than the response of the compact wet-deposited WS_2_ film (black dashed line and right ordinate, Figure 3a) under the same conditions. Similar superiority of the porous film is confirmed over a NO_2_ concentration range from 50 to 2000 ppb (Figure 3b). As film thickness and constituent nanoparticles are identical for both films, the higher response with increasing porosity is attributed to the enhanced mass transfer of NO_2_ in open film architectures with higher accessible specific surface area. In fact, the large pores in the porous film, with diameters of up to about 1 μm (Figure 1a), should facilitate rapid gas transport, even into the lower (electrode-near) regions of the film. The higher porosity of the WS_2_ nanosheets also increases the exposure of surface edges, which are typically more reactive due to higher surface energy and unsaturated bonds.^41^ Similar effects of the film morphology on sensing performance have been observed with CuBr films for ammonia sensing.^35^

**Figure 3:**
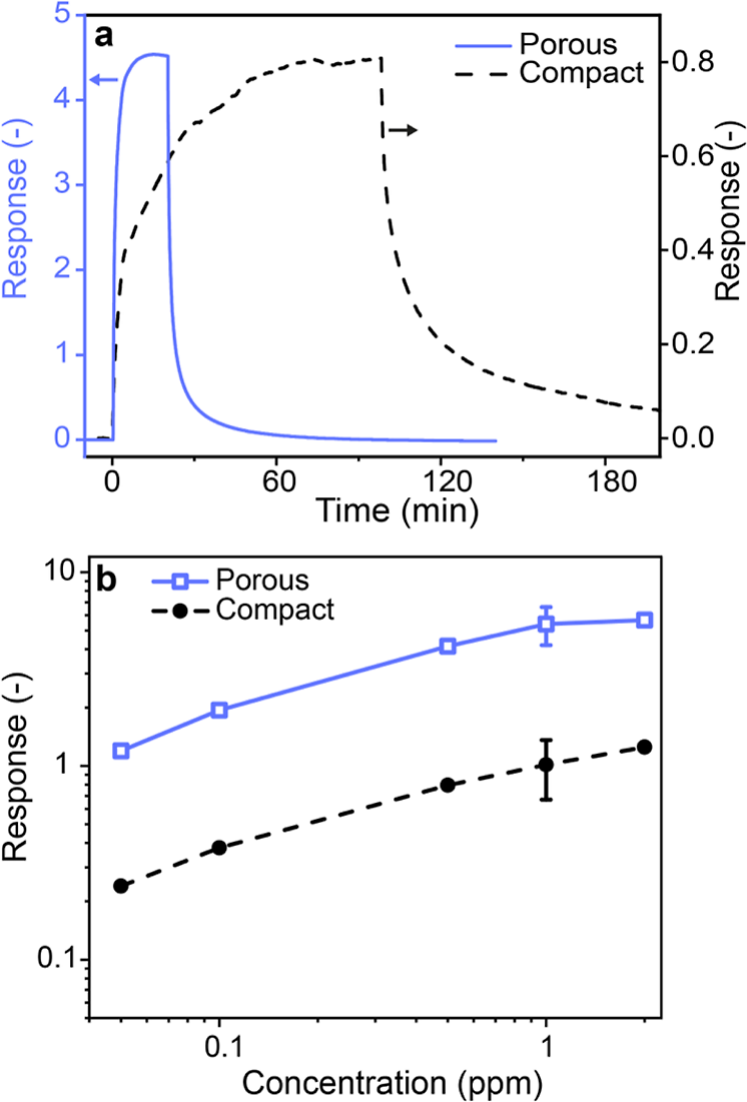
(a) Response over time of a porous (blue solid line) and compact (black dashed line) WS_2_ film to 1 ppm of NO_2_. Note the different ordinate scales. (b) Sensing response of porous (blue empty squares) and compact (black filled circles) WS_2_ films upon exposure to 50–2000 ppb of NO_2_. Symbols and error bars at 1000 ppb represent the mean ± standard deviation of six porous and two compact sensors fabricated under identical conditions. All measurements were performed at room temperature and 50% RH.

Remarkably, film porosity also strongly affects the kinetics of the sensor response. In fact, the response time to 1 ppm of NO_2_ is only 60 s for the porous film (Figure 3a, solid blue line), while it is 31 min for the more compact film (dashed black line). The performance of the porous film was comparable to, if not faster than, WS_2_ sensors described in the literature, which typically feature a more compact film morphology. For a WS_2_ sensor operated at 250 °C,^22^ a response time of 3 min was reported for 1 ppm of NO_2_, while a room-temperature WS_2_ sensor^42^ showed a response time of 4 min for 10 ppm of NO_2_, both in dry air. The recovery times of the sensors are 32 min for the porous film and 112 min for the compact film. Importantly, these response and recovery times are sufficiently fast for diurnal NO_2_ assessment at critical locations (e.g., traffic hotspots, industrial areas and energy facilities). For instance, roadside measurements on a major urban road in the U.S. showed that morning NO_2_ concentrations increase over 3–4 h, reflecting typical rush-hour traffic patterns.^43^ For applications requiring faster detection of short-term NO_2_ spikes, triggered-based sampling,^47^ UV light irradiation,^44^ heating of the sensor,^45^ or catalytic surface modifications with noble metal clusters^46^ may be applied.

### NO_2_ Sensing at Low Parts-per-Billion Concentrations and Stability

Given the revised WHO exposure limit of ∼5 ppb,^4^ the porous WS_2_ sensor was challenged for the consecutive detection of NO_2_ concentrations ranging from 500 ppb down to 1 ppb at room temperature and 50% RH in Figure 4a. Importantly, NO_2_ is quantified accurately at 1 ppb with a high signal-to-noise ratio (SNR) of 12.9, that allows clear discrimination from other concentrations (e.g., 5 ppb). The extrapolated (theoretical) limit of detection is 230 parts-per-trillion considering a minimum SNR of 3. As a result, our porous WS_2_ films reach a lower detection limit compared to previously reported chemoresistive NO_2_ sensors operated at room temperature (Table 1). Among the pristine WS_2_ sensors listed, the lowest limit of quantification is 100 ppb,^19^ while a WS_2_/ZnS heterostructure,^48^ fabricated by liquid-phase exfoliation and drop-casting, achieved 10 ppb. For a WS_2_/rGO heterostructure,^49^ a detection limit of 20 ppb was reported, whereas reduced graphene oxide (rGO)-metal oxide composites listed in Table 1 only reached quantification limits of about 50 ppb.^12,15^ Even aerosol-deposited sensors of WO_3_ ^8^ and Cu_3_N^36^ with similarly high porosities (97 and 91%, respectively) only detected down to 3 and 50 ppb NO_2_ when operated at 125 and 75 °C, respectively. Incomplete recovery after NO_2_ exposure is one of the key limitations of WS_2_ sensors operated at room temperature,^19–22,50^ primarily due to insufficient thermal energy to fully desorb the analyte molecules.^51^ In contrast, our porous WS_2_ sensor (Figure 4b) recovers the baseline after consecutive NO_2_ exposures to 1 ppm, indicating a fully reversible analyte–surface interaction. Note that six different sensors were used for the measurements, which accounts for the variation in baseline resistances, ranging from 3 to 55 MΩ (mean ± standard deviation: 33.1 ± 18.7 MΩ) across different sensors.

**Figure 4:**
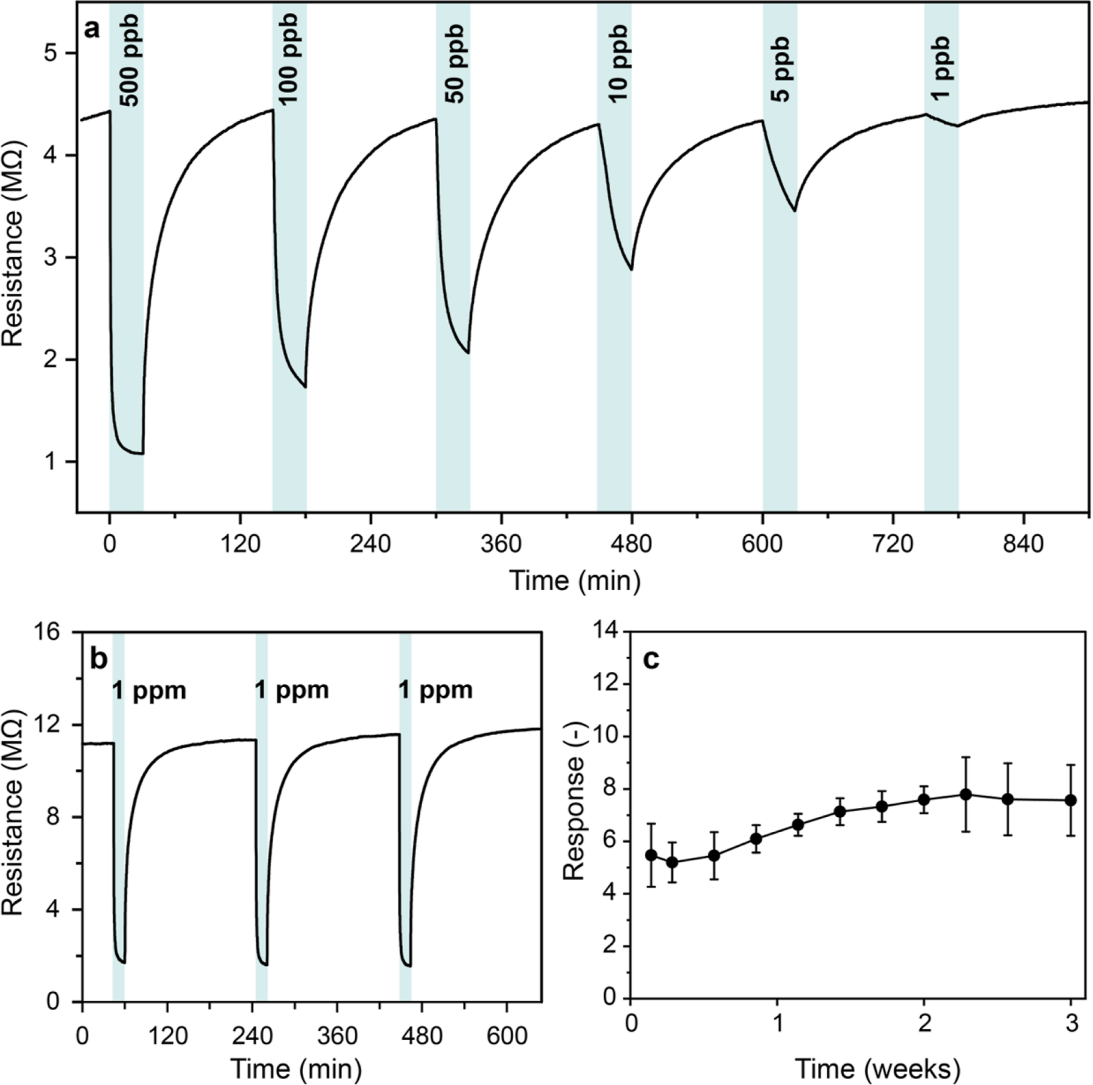
(a) Sensor film resistance upon exposure to 1–500 ppb NO_2_. (b) Sensor film resistance upon three consecutive exposures to 1 ppm of NO_2_. (c) Stability of sensor response to 1 ppm of NO_2_ for *n* = 3 sensors over 3 weeks. Indicated are the mean ± standard deviation at each time point. All measurements were performed at room temperature and 50% RH.

**Table 1:** Performance of Room-Temperature NO_2_ Sensors Based on Metal Oxide Composites (Top), Metal Sulfide Composites (Middle) and Pristine Metal Sulfides (Bottom)^a^

					NO_2_ selectivity											
material composition		fabrication method	LOQ (ppb)	RH range (%)	NH_3_	NO	Ace	H_2_S	CO	N_2_O	benzene	toluene	MeOH	EtOH	stability (% response change over time)	refs
metal oxide composites	SnO_2_/ZnO	hydrothermal and precipitation method	200	30				>10^3^	>10^5^*					>10^5^*		^60^
	rGO–SnO_2_	hydrothermal and chemical solution deposition	50	20–80	190*	3.2						223*		245*		^12^
	rGO–Fe_2_O_3_	electrospinning and calcination	100	25	3	2.2			2.9							^13^
	rGO-CNT–SnO_2_	hydrothermal method and drop-casting	1000	25	38				77							^14^
	rGO–CuO	hydrothermal method and thermal treatment	50	25–85	79										±3% in 30 days	^15^
metal sulfide composites	rGO–MoS_2_–Ag	hydrothermal synthesis, drop-casting and Ag reduction	1000		28*				>10^3^*							^50^
	WS_2_/ZnS	liquid-phase exfoliation and drop-casting	10	20–70	>10^3^*		>10^4^*					>10^3^*	>10^4^*	>10^3^*	–10% in 2 weeks	^48^
	WS_2_/rGO	hydrothermal method	2000	55–70	8.2			12		26					–13% in 2 months	^61^
	WS_2_/rGO	solution-based synthesis of van der Waals heterojunctions	20	50–60	39*			19*	150*							^49^
	WO_3_/WS_2_	hydrothermal synthesis and thermal annealing	2000	26–52	30			7		19					–14% in 100 days	^62^
	WO_3_/WS_2_	hydrothermal method and calcination	500	30–90	6*				178*					14*	–15% in 30 days	^63^
	WS_2_/SnO_2_	chemical exfoliation and self-assembly	50	0–80	8.5			11	19					5	–2% in 30 days	^64^
pristine metal sulfides	NbS_2_	spin-coating and thermal chemical vapor deposition	240	10–50	65*		562*				409*			514*	–35% in 3 months	^65^
	WS_2_	hydrothermal method and calcination	100	23–95												^19^
	WS_2_	freeze-drying aerogel synthesis	200	0	1.2*											^22^
	WS_2_	chemical vapor deposition	400	0–80	6*		74*	322	44*						–20% in 2 weeks	^20^
	WS_2_	flame-aerosol deposition and dry-conversion	1	0–90	164	217	361	>10^3^	>10^3^	>10^3^	>10^3^	>10^3^	>10^3^	>10^3^	±10% for >6 months	this work

a Material composition, fabrication method, limit of quantification (LOQ), tested RH range, selectivity towards NH_3_, NO, acetone (Ace), H_2_S, CO, N_2_O, benzene, toluene, methanol (MeOH) and ethanol (EtOH) and long-term stability (percentage response change over time) are indicated. The LOQ represents the lowest measured concentration. Selectivity was determined at same analyte concentration. Linear response-concentration characteristic was assumed in case responses had to be extrapolated, as indicated by *-symbols.

Furthermore, the long-term stability over 3 weeks was tested for three identically produced sensors (Figure 4c). The average response toward 1 ppm of NO_2_ shows good stability between 5.2 and 7.8 (error bars indicate standard deviation), without any continuous degradation. During the first week after sensor fabrication, the sensor response was slightly lower, which could be explained by surface stabilization effects or residual gases from the sulfidation process. For one additional sensor, the long-term stability was assessed over more than 6 months with a stable response (Figure S4).

### Selectivity and Humidity Robustness

Robustness against interfering analytes as well as varying RH is critical for a NO_2_ sensors in real-world applications (e.g., air quality monitoring). Therefore, the porous WS_2_ sensor was tested to a variety of air quality-relevant reducing and oxidizing analytes from different chemical classes at 1 ppm and 50% RH (Figure 5a): NH_3_, NO, acetone, H_2_S, benzene, CO, ethanol, methanol, N_2_O and toluene. The sensor almost exclusively responds to NO_2_ yielding selectivities over all other analytes ≥ 164 for NH_3_ and even >1000 for alcohols or aromatic compounds. To further validate selectivity under more realistic conditions, we additionally compared the sensor response for 50 ppb NO_2_ against orders of magnitude higher concentrations of NH_3_, ethanol and CO in Figure 5b. Even at 250,000 ppb ethanol and CO, the responses remained negligible (0.02 and 0.005, respectively). These results confirm that the porous WS_2_ films retain high selectivity toward NO_2_ even when common interferents are present at significantly higher concentrations, as relevant for air quality monitoring. As a result, it outperforms other pure WS_2_ sensors (Table 1), which showed significant interference, for instance, by NH_3_ (selectivity ≤ 6 ^20,52,53^ ). High selectivity for NH_3_ is crucial because it can be present at significantly higher concentrations in air, particularly from agricultural emissions.^54^ An investigation of binding energies between the gas molecule and the WS_2_ surface showed that basal planes favor the adsorption of reducing analytes (e.g., NH_3_), whereas edge sites interact stronger with oxidizing analytes (i.e., NO_2_).^51,55,56^ This may explain the observed higher selectivity toward NO_2_ compared to WS_2_ sensors produced by other methods, as FSP combined with dry sulfidation yields nanosheets of varying sizes (Figure 1a) and many available edge sites due to the high porosity.

**Figure 5:**
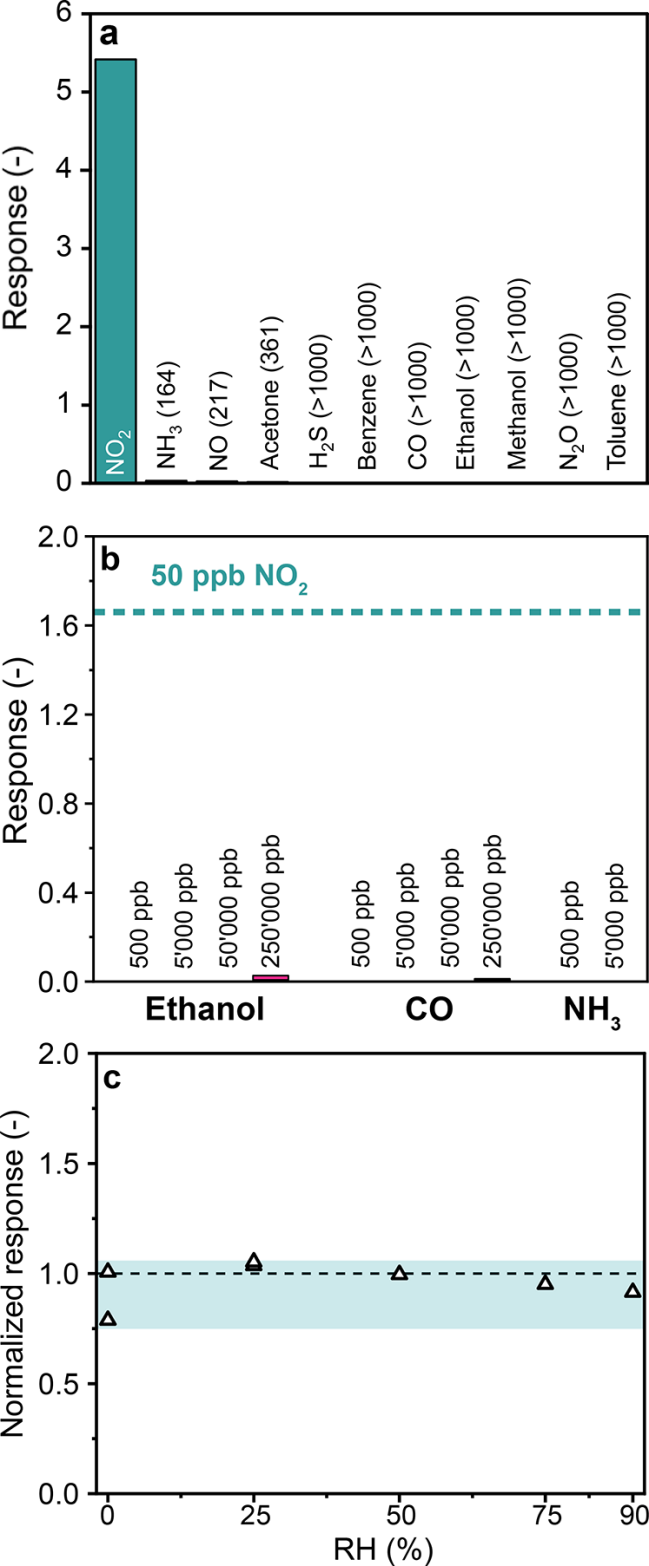
Sensor response of porous WS_2_ film against air quality-relevant interferents and varying RH. (a) Response to 1 ppm of various analytes at room temperature and 50% RH. (b) Sensor response to 500, 5000, 50,000 and 250,000 ppb of ethanol and CO, and 500 and 5000 ppb of NH_3_. The response to 50 ppb NO_2_ is indicated as a dashed line for reference. (c) Effect of RH on sensor response to 1 ppm of NO_2_. For each RH level, two NO_2_ pulses were measured on different days. The shaded area indicates the 95% confidence interval.

Finally, we tested our porous WS_2_ sensor to 0–90% RH. Figure 5c shows the response to 1 ppm of NO_2_ (normalized to the response at 50% RH). Importantly, the response remains stable within a 95% confidence interval from 0.75 to 1.06 across the tested RH range. Specifically, the response decreases only by 8.4% when increasing the RH from 50 to 90%. This is superior to most previously reported WS_2_ sensors operated at comparable temperatures that showed a significant response reduction at high RH.^19,37,52^ In WO_3_, NO_2_ sensing is based on interaction with surface oxygen vacancies. Since water molecules oxidize these oxygen vacancies, increasing relative humidity reduces sensor performance.^57^ In contrast, experimental reports indicate that NO_2_ interacts with WS_2_ by physisorption and surface charge-transfer,^58^ rather than through vacancy chemistry. Consequently, the NO_2_ sensing mechanism on WS_2_ should be robust to relative humidity changes, in agreement with our observation in Figure 5c.

## Conclusion

We demonstrated a dry synthesis approach combining flame-aerosol deposition with dry sulfidation to fabricate nanostructured WS_2_ films with unprecedented high porosity (98 ± 1%). The porous WS_2_ films exhibit a 5-fold increase in response and more than an order-of-magnitude faster response time to 1 ppm of NO_2_ compared to compact spin-coated films at room temperature and 50% RH. These enhancements are attributed to the higher accessible surface area and improved gas diffusion through the interconnected porous network. Most notably, the sensor achieves a (demonstrated) limit of quantification as low as 1 ppb NO_2_ with an SNR of 12.9. High selectivity is demonstrated against various interfering gases, including NH_3_, NO, acetone, CO and H_2_S, with some tested even at orders of magnitude higher concentrations. Finally, the sensor maintains excellent performance across a wide humidity range (0–90% RH) and exhibits long-term operational stability over at least six months. These findings highlight the potential of flame-made and dry-sulfidized, porous WS_2_ films for integration into room-temperature sensor platforms for low-power applications. Their robustness and sensitivity make them highly suitable for deployment as hand-held^59^ air-quality monitors in industrial and agricultural settings, as well as for use in personal exposure monitoring and large-scale environmental sensing networks.

## Supplementary Material



## Data Availability

The data that support the findings of this study can be requested from the corresponding author.

## Notes

Schematic of experimental gas sensing setup, cross-sectional SEM images of WS_2_ films of various thicknesses, discussion of XPS O 1s spectra of WO_3_ and WS_2_, and 6 month stability assessment (PDF)
