# Supplementary material for: Selective, Stable and Humidity-Robust NO2 Sensing at Room Temperature with Porous WS2 Films
Source: ACS Sens. 2025 Oct 27;10(11):8596–605. doi: 10.1021/acssensors.5c02414 (PMC12670997; doi:10.1021/acssensors.5c02414)
Supplement: Supplementary file 1 [file se5c02414_si_001.pdf]

Supplementary Information

Selective, Stable and Humidity-Robust NO_2 Sensing at Room Temperature with Porous WS_2 Films

Simone Hersberger[1], Michael Pereira Martins[1], Selina Fassbind[1], Andreas T. Güntner[1,*]

[1]Human-centered Sensing Laboratory, Department of Mechanical and Process Engineering, ETH Zurich, CH-8092 Zurich, Switzerland

*corresponding author: andregue@ethz.ch

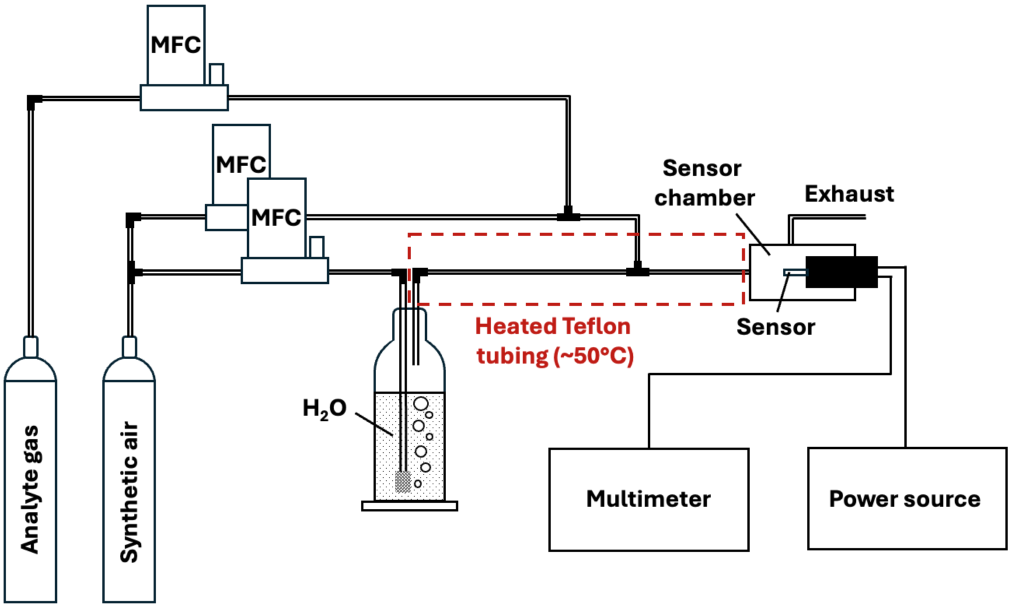

Figure S1: Schematic of experimental setup for gas sensing. The analyte gas from a certified gas standard was mixed with synthetic air using high-precision and calibrated mass flow controllers. Relative humidity was introduced by bubbling dry synthetic air through deionized water at 22 – 23 °C. The sensors were mounted onto a Macor holder inside a Teflon chamber and the film resistance was monitored between the interdigitated Pt electrodes of the sensor substrate using a multimeter.

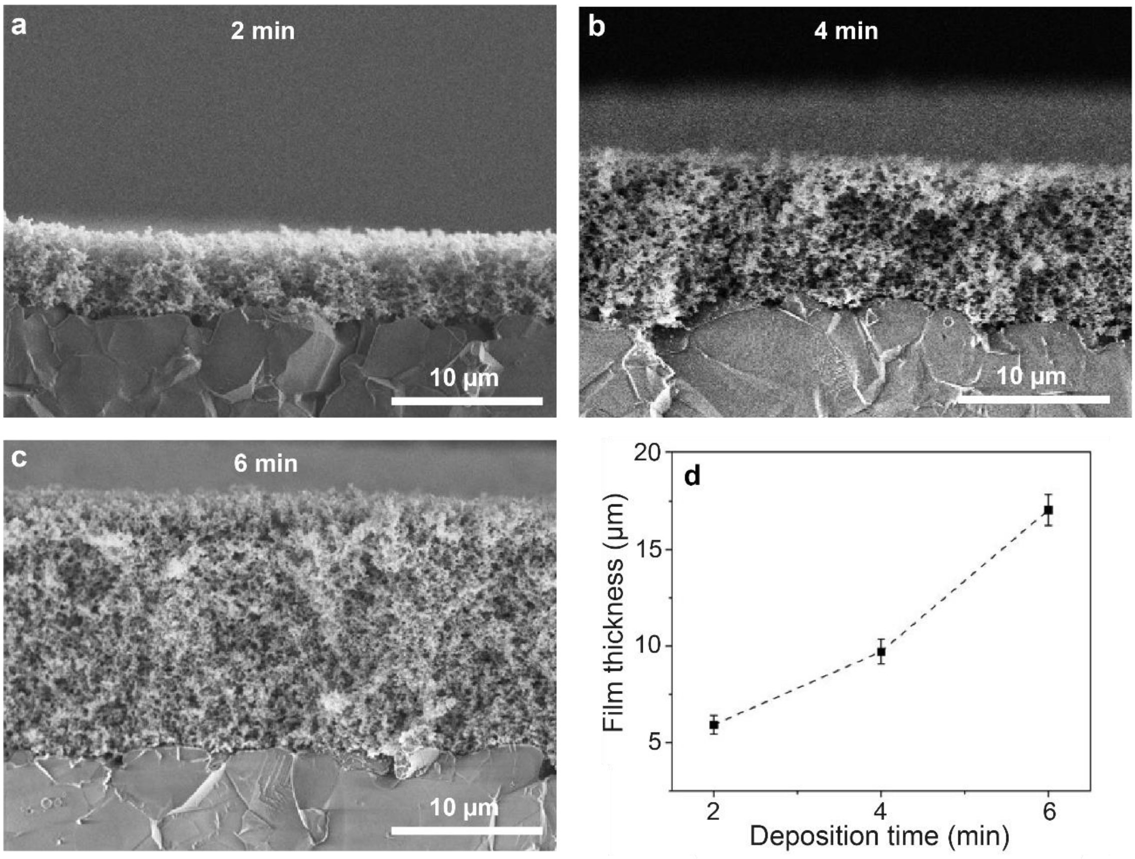

Figure S2: Cross-sectional SEM images of WS_2 films, produced by aerosol-deposition of WO_3 for (a) 2 min, (b) 4 min and (c) 6 min, followed by dry sulfidation. (d) Film thickness as a function of deposition time. Indicated are the mean ± standard deviation of >100 measurements per film.

High-resolution XPS O 1s of WO_3 and WS_2

High-resolution O 1s spectra were collected for pristine WO_3 and the dry-sulfidized WS_2. The WO_3 spectrum was deconvoluted into three components[66]: lattice oxygen (O_L) at 529.7 eV (W–O bonds), chemisorbed oxygen (O_C) at 531.7 eV (surface –OH or defect-related species) and physisorbed oxygen (O_{Ph}) at 533.0 eV (adsorbed H_2O or O_2). In contrast, the WS_2 spectrum shows no detectable O_L peak and can be satisfactorily fitted only with O_C at 531.5 eV and O_{Ph} at 535.3 eV. The absence of the characteristic lattice oxygen signal from WO_3 suggests that no residual tungsten oxide remains on the WS_2 surface.

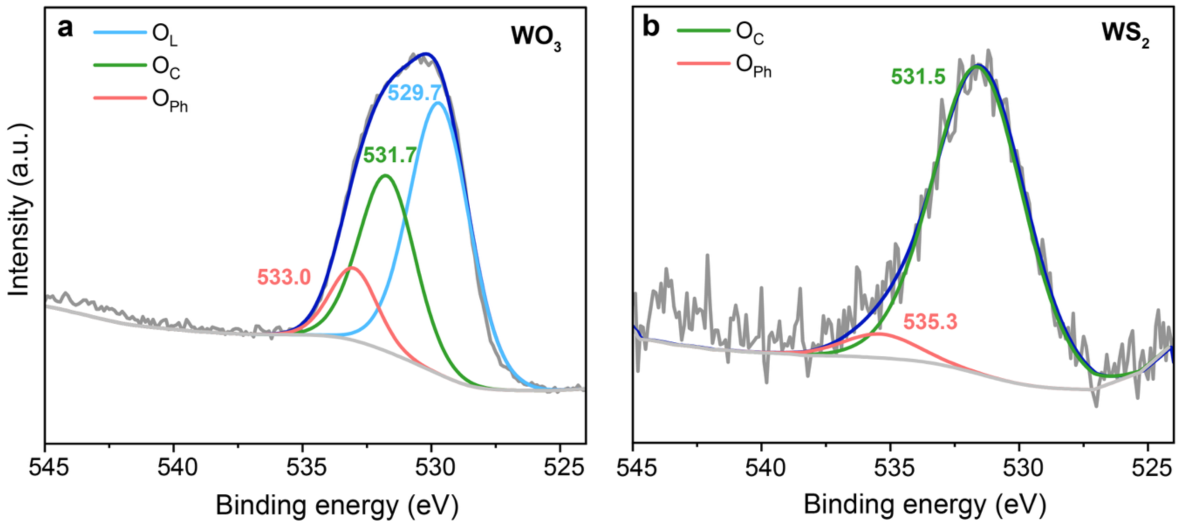

Figure S3: High-resolution XPS O 1s spectra of (a) pristine WO_3 and (b) dry-sulfidized WS_2 powders. The WO_3 spectrum was deconvoluted into lattice (O_L), chemisorbed (O_C) and physisorbed oxygen (O_{Ph}), whereas the WS_2 spectrum can be fitted only with O_C and O_{Ph}.

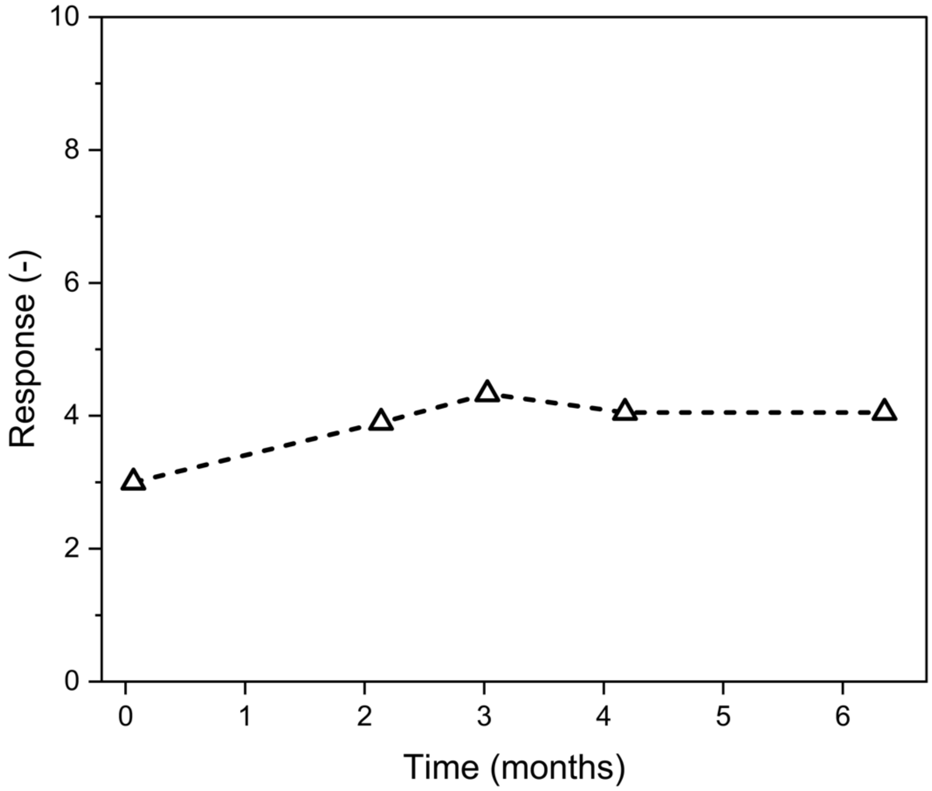

Figure S4: Long-term stability of sensor response to 1 ppm NO_2 over more than 6 months. All measurements were performed at room temperature and at 50% RH.

References

66. Frankcombe, T. J. & Liu, Y. Interpretation of Oxygen 1s X-ray Photoelectron Spectroscopy of ZnO. *Chem. Mater.* **35**, 5468–5474 (2023).
